# Unasur Health Council: An emerging global actor in health policy and governance

**DOI:** 10.1177/1468018115600123c

**Published:** 2015-12

**Authors:** Mariana Faria

**Affiliations:** International Relations Coordinator of the South American Institute of Government in Health (ISAGS/UNASUR)

In 2005, heads of State from South America with progressive tendencies expressed their rejection to the creation of the Free Trade Area of the Americas (FTAA) and began a new project that aimed to achieve alternative modes for regional governance, which resulted in the creation of the Union of South American Nations (Unasur). Unasur has been established as an alternative integration project, with renewed commitments about the principles of democracy, social inclusion and human rights.

In this context, health has become an important field of integration and promotion of the right to health based on universality, equity and integrality. With the creation of the Health Council, Unasur is starting to implement health projects, fostering the exchange of knowledge and generating regional strategies to support the coordination of common positions for countries in multilateral forums. In all these ways, Unasur is helping to strengthen the development of this regional bloc as a global player in the field of health governance and policy.

## Unasur and the Health Council

Unasur is formed by the 12 independent countries of South America. Its vertical structure is formed by the councils of heads of State, ministers of foreign affairs, national delegates and a General Secretariat. The horizontal structure counts with 12 sectorial ministerial councils that deal with specific issues such as infrastructure, education, defence, health and others. Unasur also counts with two strategic thinking centres, one that is part of the Defence Council and the other one, part of the Health Council, the South American Institute of Government in Health (ISAGS-UNASUR).

The South American Health Council (SHC) was created in 2008 from the consensus that health is a fundamental right of the human being and of the society. Its objectives are consolidating South America as a space for integration in health, contributing to everyone’s health and development, incorporating and integrating sub-regional efforts and achievements (UNASUR, 2008).

Since its creation, the SHC has organised eight ordinary meetings, in which the ministers of health have debated and decided about issues such as the H1N1 epidemics, the election for the direction of the PAHO and the creation of a regional medicine price database (UNASUR, 2012).

## Unasur at the World Health Assembly

Besides its regional actuation, the SHC’s actions are aimed at projecting its objectives in diverse spaces of global health governance, not just within South America but beyond, in multilateral forums’ governance. For example, the actuation of the SHC at the World Health Assembly (WHA) started in 2010, the year in which Unasur approved the SHC Five-Year Plan, and it establishes a milestone in the history of the South American representation in the scenario of global health policy making. The SHC works prior to the discussions in order to reach consensus regarding a specific issue. The elaboration of common positions can take place in in-person meetings as well as in virtual meetings. The in-person meetings take place during the ordinary and extraordinary meetings of the council of ministers and coordinating committee, especially in ordinary meetings held during the first semester, before the WHA, or in the extraordinary meetings held in Geneva, during the WHA. Besides the SHC meetings, meetings of the TGs, Workshops hosted by ISAGS-UNASUR and other multilateral forums are used by Unasur in order to gather together and debate issues that in the future will be presented at the WHA as a common position of the bloc. Another privileged space for the concerted action of the SHC is the WHO Executive Board. Since 2013, three of the six representatives of the Americas on the Executive Board are Unasur Member Countries: Argentina, Brazil and Suriname. The Coordinating Committee and the TGs also organise several virtual meetings throughout the months preceding the WHA, in order to have detailed discussions about the texts of draft resolutions and common interventions (Ventura, 2013).

There are two entryways for issues that are put forward for debate and for consensus within the bloc: they can come from one of the SHC structures, such as the TG, or they can be presented by a country, which has a determined issue as a national priority and pursues the necessary support among its partners in the bloc in order to move forward with the issue in the global scenario. The construction of a regional health agenda can happen through both mechanisms, given that, once the countries agree to embrace the issue of one of the members of the bloc as a regional priority, all 12 countries are equally responsible for it from that moment on.

During the last WHA, held in May 2015, one of the most relevant topics of the agenda was the position of Health in the Post-2015 Development Agenda. Unasur presented a common position on that. The 12 countries of South America manifested that besides recognising the importance of the general goal to ‘Ensure healthy lives and promote wellbeing for all at all ages’, as well as its 9 health targets, it is fundamental to highlight the need for intersectoral work, for the enjoyment of the highest attainable degree of health.

Unasur Health Council acknowledged the responsibility of the health sector in the linkage with other goals of the post-2015 agenda and the importance of keeping an integral perspective, with a multisectoral approach, aiming to reduce inequalities towards development and considering that the social determinants are a pillar that is present in health policies of Unasur countries.

Unasur is a new regional bloc ‘under construction’, and over the coming years it has significant ambitions and opportunities to intensify its work as a global actor. Three principal challenges arise.

The first challenge is the informality of Unasur’s representation at the WHA, which still limits its actuation. The recognition of the bloc as an observer with a seat in the WHA can be the next step for the consolidation of Unasur as a player in the health arena.

Unasur represents only 12 votes in a universe of 194 votes in total. Therefore, it should work on building alliances with other regions and countries that can strengthen the positions and values defended by the bloc and gather votes. The Global South should be the main partner, especially with other blocs of countries such as the African Union, Arab League, BRICS, Caricom, Comisca, Community of Portuguese-speaking Countries (CPLP) and SADC. However, the health diplomacy developed by Unasur can also incorporate countries from Europe and Canada, for example, considering the values and principles that transversely appear in their common positions regarding global health issues.

A second challenge is to generate data and evidence about regional priorities that can underpin the development of common positions. It is necessary to learn from great global players such as countries from the North and institutions like the World Bank, which use data to convince and present consistent information based on numbers, with the development of technical capacity and the strengthening of their negotiation ability.

The strengthening of the region through intersectoral policies and actions is important for the growth of Unasur’s actuation in the global arena, beyond the field of health. Unasur counts with other 11 Ministerial Councils, besides health, and a Council of Ministers of Foreign Affairs. Once this structure starts to coordinate its actions and to work conjointly in different global arenas, the bloc will be able to strengthen its position as a global player.

The third challenge is for the SHC and Unasur to come closer to civil society and the South American academy. The establishment of bonds with social movements and with the academy is an important step for the construction of a new democratic health governance in the region. These regional players have been fighting for health and for democracy over decades and therefore should be Unasur allies.
